# From Waste to Resource: Extraction and Characterization of Polyphenols from Dalmatian Olive Mill Wastewater

**DOI:** 10.3390/antiox15010012

**Published:** 2025-12-21

**Authors:** Nina Knezovic, Ajka Pribisalic, Katarina Jurcic, Ivica Ljubenkov, Barbara Soldo, Danijela Skroza, Mladenka Sarolic, Sanja Luetic, Davorka Sutlovic, Zlatka Knezovic

**Affiliations:** 1 Teaching Institute for Public Health, Split-Dalmatia County, 21000 Split, Croatia; 2 Faculty of Health Sciences, University of Split, 21000 Split, Croatia; 3 Department of Public Health, School of Medicine, University of Split, 21000 Split, Croatia; 4 Department of Chemistry, Faculty of Science, University of Split, Ruđera Boškovića 33, 21000 Split, Croatia; 5 Department of Food Technology and Biotechnology, Faculty of Chemistry and Technology, University of Split, R. Boškovića 35, 21000 Split, Croatia; 6 Department of Applied Pharmacy, School of Medicine, University of Split, 21000 Split, Croatia

**Keywords:** polyphenols, olive mill wastewater, polyphenol profile, HPLC, antioxidant activity, antioxidants

## Abstract

Background: Olive cultivation and olive oil production are key agricultural sectors in the Dalmatia region, where numerous oil mills operate. Analyses have shown that extra virgin olive oils (EVOO) produced in this area contain respectable amounts of polyphenols, which contribute to superior oil quality due to their antioxidant properties. During processing, hydrophilic phenolic compounds predominantly transfer into olive mill wastewater (OMW), making it a concentrated source of valuable bioactive molecules. The antioxidant, anti-inflammatory, and photoprotective effects of these polyphenols are highly relevant for cosmetic and pharmaceutical use. Methods: A total of 186 OMW samples were collected from oil mills in the Split-Dalmatia County across three production seasons (2023–2025). Total polyphenol content (TPC) was measured spectrophotometrically, while polyphenol composition was determined by High Performance Liquid Chromatography (HPLC). Antioxidant activity was evaluated using hydrogen atom transfer (HAT; 2,2-diphenyl-1-picrylhydrazyl) (DPPH), electron transfer (ET; ferric reducing antioxidant power) (FRAP), and oxygen radical absorbance capacity assay (ORAC). Results: The obtained results indicated high total polyphenols concentrations, with values ranging from 111.8 to 6717.2 mg of gallic acid equivalents per L of OMW (mg GAe L^−1^). In the vast majority of analyzed samples, hydroxytyrosol was the predominant phenol compound. The antioxidant activity of the samples was high.

## 1. Introduction

Polyphenols, classified as phytochemicals with phenolic groups, occur abundantly in plants [1] and exhibit antioxidant, anti-inflammatory, and antimicrobial properties that support human health [2,3,4]. Extra virgin olive oil (EVOO) contains over 30 polyphenolic compounds [5], primary phenolic acids (e.g., hydroxybenzoic, p-coumaric, ferulic, gallic, syringic, vanillic, caffeic, and o-coumaric), secoiridoids (e.g., demethyloleuropein, oleuropein, ligstroside, and their derivatives oleacein and oleocanthal), as well as phenolic alcohols (e.g., hydroxytyrosol and tyrosol) [5,6,7].

Secoiridoids like oleocanthal and oleacein, formed via enzymatic degradation of oleuropein and ligstroside during EVOO processing, show strong anti-inflammatory and neuroprotective effects [8,9]; oleuropein predominates in unripe olives but declines with ripening, while hydroxytyrosol and tyrosol arise from hydrolysis during ripening and storage [10,11,12].

EVOO phenolic levels vary with cultivar, cultivation methods, fruit maturity, health status, and processing techniques [13]. Due to favourable agroecological conditions and long tradition, Croatia is one of the countries producing high-quality extra virgin olive oils with high polyphenol content [14,15]. The largest areas under olive trees are located in Dalmatia, especially in the Split-Dalmatia County [16]. The method of processing olives in oil mills is of key importance for preserving the nutritional and sensory properties of the oil, especially the content of polyphenols and aromatic compounds [17]. Optimal production requires healthy fruits processed promptly post-harvest to minimize oxidation, as fly-damaged olives reduce polyphenol content and oil quality, such as poorer physical and chemical characteristics, but also with a lower content of polyphenols [18].

Unlike in the past, when hydraulic presses were the main equipment, modern Croatian production favours centrifugation systems. Post-cleaning and grinding, the malaxation phase is crucial for the quality of the oil, as well as for the content of polyphenols. In this stage, oil droplets merge, with paste temperature, process duration, and water addition affecting oil quality and phenol retention [17,18,19,20]. After malaxation, olive paste is centrifuged on a horizontal centrifuge (decanter), and the oil fraction is separated from solids (crushed olive and pit).

**Figure 1:**
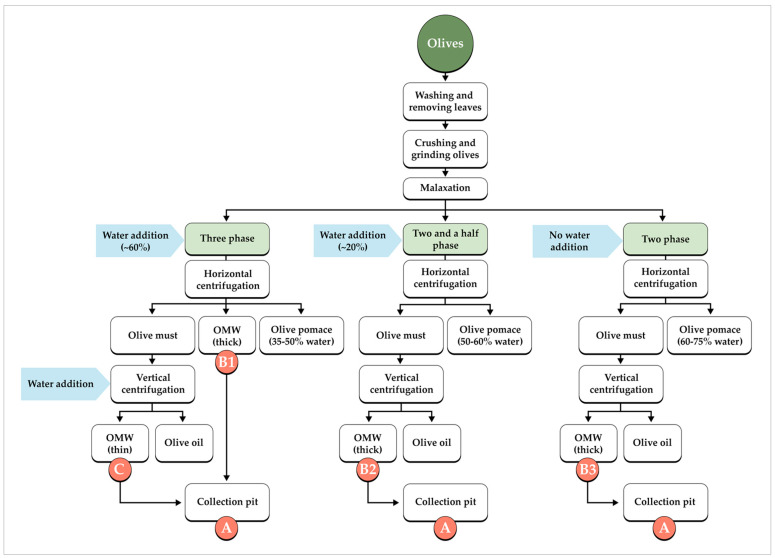
Process flow diagrams of the different olive oil extraction systems and their final product streams, sampling locations from various points in production (A—collection pit, B1—3PD after horizontal centrifugation, B2—2.5PD after horizontal and vertical centrifugation, B3—2PD after horizontal and vertical centrifugation, C—3PD after horizontal and vertical centrifugation).

There are two different types of centrifugation processes: three-phase (3PD) and two-phase decanter (2PD). In the 3PD system, approximately 50–70 L of lukewarm water is added per 100 kg of olives, yielding three separate streams after centrifugation: pomace, OMW, and olive oil. In contrast, the 2PD system operates without the addition of water to the olive paste, producing only two phases: pomace and oil. Consequently, the 3PD process generates larger volumes of OMW and pomace with lower moisture content, whereas the 2PD process produces substantially less OMW but results in pomace with a higher water content. In the Dalmatia region, two-phase horizontal centrifugation is predominant, along with a hybrid technology known as the two-and-a-half-phase process (2.5PD), combining features of the 3PD and 2PD and adding minimal water (<20 L/100 kg) [21,22,23]. These methods preserve polyphenols and sensory qualities, with 2PD and 2.5PD dominant for lower environmental impact (Figure 1).

Untreated pomace and OMW are phytotoxic and can damage soil and plants if directly discharged onto agricultural land [24]. OMW is a dark brown emulsion containing finely dispersed olive particles, sugars, lipids, organic acids, polyphenols, minerals, and salts [25]. Because many polyphenols are water-soluble, a substantial proportion partitions into the aqueous phase, where their high concentrations can inhibit the absorption of phytonutrients essential for plant growth and development [26].

At the same time, these phenolic compounds exhibit strong antioxidant, anti-inflammatory, and other bioactive properties, which make OMW a valuable but underused raw material for polyphenol recovery. Modern valorization strategies focus on concentrating and purifying OMW polyphenols for use as functional ingredients in foods, nutraceuticals, and cosmetics [25,26,27]. The aim of this research is to quantify total and individual polyphenol concentration in OMW from oil mills in the Dalmatia region and to characterize their phenolic profile. In the next phase, these data will be used to assess the feasibility of OMW as a sustainable source of bioactive polyphenols for potential cosmetic applications, taking advantage of their antioxidant and other beneficial properties.

## 2. Materials and Methods

### 2.1. Samples

A total of 186 samples of OMW were collected during the olive processing season from 2023 to 2025. Of these, 61 samples were obtained from collection pits (CP), (symbol A from Figure 1), as part of routine wastewater quality monitoring, while 125 samples were gathered from various points along production lines (symbols B1–B3 from Figure 1) operating with three-phase (3PD), two-phase (2PD), and two-and-a-half-phase (2.5PD) systems (21 from the 2023 and 2024 harvest season, and 101 from 2025 harvest season). Each OMW sample was collected in a volume of 2 L and stored at a temperature of +4 °C until analysis.

### 2.2. Sample Preparation

Three procedures were used to find a method that produced the highest total phenols. One technique without extraction, i.e., direct measurement from the samples, and two extractions using organic solvents, methanol and ethanol.

For the methanolic extraction, 30 mL of OMW were mixed with 20 mL of the methanol–water solution (80:20 *v*/*v*) and vortexed for 2 min. The mixture was then subjected to ultrasonic bath (Sonis 4GT, Iskra, Ljubljana, Slovenia) at 40 kHz for 15 min at a temperature below 30 °C. Subsequently, the samples were centrifuged for 15 min at 5000× *g* using a Tehtnica 4GT centrifuge (Iskra, Ljubljana, Slovenia), after which the supernatant was filtered first through a glass fibre syringe filter (pore size of 1.2 µm) and then through a nylon syringe filter (0.45 µm) [27].

The same procedure was applied using ethanol–water (80:20 *v*/*v*) as the extraction solvent. After centrifugation and filtration, an aliquot of each OMW extract was used directly for total phenolic content (TPC) analysis.

### 2.3. Characterization of OMW

The OMW samples were characterized using several physicochemical parameters, including pH value—HRN EN ISO 10523:2012 [28], colour—Standard Methods for the examination of water and wastewater, (24th Edition 2023, 2120 C [29], odour—Standard Methods for the examination of water and wastewater, 24th Edition 2023, 2150 B [30], suspended solids—HRN EN 872:2008 [31], chemical oxygen demand—HRN ISO 15705:2003 [32], and biological oxygen demand—HRN EN ISO 5815-1:2019 [33].

### 2.4. Determination of Total Phenolic Content (TPC)

TPC was determined spectrophotometrically using the Follin Ciocoltau method with minor modifications [34]. A 0.1 mL aliquot of the sample was transferred in volumetric flask and diluted to 5 mL with deionized water (0.05 mS cm^−1^) obtained from an Ultrapure water system (Omnia Pure, Stakpure GmbH, Niederahr, Germany). Subsequently, 0.5 mL of Follin Ciocoltau phenol reagent (Sigma-Aldrich, St. Louis, MO, USA) was added. After 3 min, 1 mL of 20% (*w*/*v*) Na_2_CO_3_ solution (Sigma-Aldrich, St. Louis, MO, USA) was added, and the volume was adjusted up to 25 mL with deionized water. The samples were kept in the dark for 60 min, after which the absorbance was measured at wavelength 760 nm using UV-VIS spectrophotometer (Lambda 25, Perkin Elmer, Shelton, CT, USA). All measurements were performed in duplicate (analytical replicates), and the TPC values per sample were calculated as mean values in mg of gallic acid equivalents per litre (mg GAe L^−1^) of OMW. Due to non-normal distribution of biological replicates (Shapiro–Wilk test), dataset results were expressed as median with interquartile range (IQR) and concentration range (mg GAe L^−1^). The calibration curve was constructed using gallic acid standard solutions (Sigma-Aldrich, St. Louis, MO, USA) at concentrates ranging from 5 to 20 µg mL^−1^, yielding with a linearity (R^2^) of 0.999.

### 2.5. Characterization of Phenolic Content

High Performance Liquid Chromatography (HPLC) analysis was performed directly on OMW without prior solvent extraction. Samples were centrifuged at 5000× *g* for 15 min and subsequently filtered through syringe filters (glass with a pore size of 1.2 µm; nylon 0.45 µm; PTFE 0.45 µm).

Phenolic compounds were separated by HPLC according to the COI/T.20/Doc. No 29/Rev.2/2022 [35]. Analyses were carried out on HPLC system (Perkin Elmer Series 200, Perkin Elmer, Waltham, MA, USA), equipped with UV/VIS detector set at 280 nm and an Ultra Aqueous C18 column (250 × 4.60 mm) with a stationary phase diameter of 5 µm (Restek, Bellefonte, PA, USA). The mobile phase consisted of 0.2% phosphoric acid in water (solvent A) and a 1:1 mixture of methanol and acetonitrile (solvent B).

Gradient elution started at 4% and was increased linearly to 50% over 40 min, then further increased to 60% over the next 5 min, reaching 100% from 45 to 60 min. After isocratic elution at 100% for 8 min, solvent B was decreased steeply to 4% between 68 and 70 min and held constant for the last 10 min to ensure stabilization of the column to the initial conditions. The column was thermostated at 25 °C, the flow rate was 0.8 mL/min, and the injection volume was 20 µL.

### 2.6. Determination of Antioxidant Activity

For the determination of antioxidant activity, OMW samples were centrifuged at 5000× *g* for 15 min and filtered subsequently through glass (pore size of 1.2 µm) and nylon (0.45 µm) syringe filters. Next, 10 mL of the filtered sample was washed with 10 mL of hexane to remove residual oils. After separation from hexane, the sample was evaporated to dryness under a vacuum evaporator (IKA RV 10, Breisgau, Germany) at 24 mbar with a controlled water bath temperature of 30 ± 1 °C. The dried extract was re-dissolved in 96% ethanol and further diluted to a working concentration of mg/mL for antioxidant assay.

The antioxidant activity was evaluated using three complementary assays reflecting different mechanisms: hydrogen atom transfer (HAT; 2,2-diphenyl-1-picrylhydrazyl (DPPH) and electron transfer (ET; ferric reducing antioxidant power (FRAP) assay) as well as oxygen radical absorbance capacity (ORAC) assays. All assays were conducted in triplicate in 96-well microplates using a Sunrise microplate reader (Tecan, Männedorf, Switzerland). Results were expressed as millimole of Trolox equivalents (mM TE) and reported as mean ± standard deviation.

#### 2.6.1. DPPH Assay

The free radical scavenging capacity of the extracts was determined using the DPPH assay [36]. A DPPH stock solution (4 mg/100 mL in ethanol) was prepared and diluted to achieve an absorbance of 1.2 ± 0.02 at 517 nm. For each measurement, 290 µL of the DPPH solution was pipetted into the wells of the microtiter plate, and the initial absorbance was recorded. Subsequently, 10 µL of the sample (diluted 1:100) was added, and the decrease in absorbance was measured after 1 h.

#### 2.6.2. FRAP Assay

The reducing activity of the extracts was determined using the FRAP assay, modified for microtiter plate format [36]. Briefly, 300 µL of FRAP reagent was pipetted into each well, and the initial absorbance was measured at 592 nm. Then, 10 µL of the sample was added, and the absorbance change was observed after 4 min.

#### 2.6.3. ORAC Assay

The ORAC assay was employed to assess the antioxidant activity based on the inhibition of peroxyl radical-induced oxidation [36]. Briefly, 150 µL of fluorescein solution and 25 µL of the sample (Trolox standard or blank) were added to the wells of the microtiter plate and incubated at 37 °C for 30 min. The reaction was initiated by adding 25 µL of AAPH (2,2′-Azodiisobutyramidine dihydrochloride) solution, and the decrease in fluorescence intensity was recorded over 90 min.

#### 2.6.4. Statistical Analysis

Normality of data distribution was assessed using the Shapiro–Wilk test. Due to non-normal distribution (*p* < 0.05), non-parametric tests were applied. Differences between multiple independent groups were analyzed using the Kruskal–Wallis test followed by Dunn’s post hoc test with Holm and Bonferroni correction. Paired sample comparisons were performed using the Wilcoxon signed-rank test or Friedman test, followed by Conover’s post hoc test with Holm correction. Statistical significance was set at *p* < 0.05. Analyses were conducted using JASP (Version 0.95.4; JASP Team, 2025).

## 3. Results and Discussion

In the results and discussion section, the sampling locations of the OMW samples are referred to using the designations presented in Figure 1 (A–C).

### 3.1. Characteristics of OMW Samples

The OMW samples exhibited acid pH values (4.7–6.0), a pronounced dark colour (2216–7065 Pt-Co mg L^−1^), and an intensive odour (1400–10,000 TON). The samples also had a high suspended solids content (3967–66,181 mg L^−1^), elevated chemical oxygen demand (COD, 4090–64,800 mg O_2_ L^−1^), and biological oxygen demand (BOD, 1600–11,000 mg O_2_ L^−1^), indicating a substantial organic load. Furthermore, the considerable concentration of total oils and fats, in combination with the low pH, confirms that such wastewater requires appropriate treatment before discharge into natural recipients (water bodies or soil) to minimize its impact on biological processes [37].

### 3.2. Total Phenol Content (TPC)

The analysis revealed that the studied OMW samples contained high concentrations of total phenols. Table 1 presents the total phenolic content expressed as gallic acid equivalent (GAe) following extraction with 80% (*v*/*v*) methanol and 80% (*v*/*v*) ethanol. As the data were not normally distributed (Shapiro–Wilk test; W(46) = 0.82, *p* < 0.001), the results are reported using median, interquartile (IQR), and concentration range (minimum–maximum observed values).

**Figure 2:**
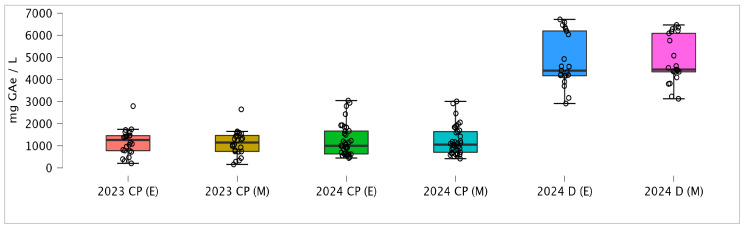
Concentrations of total phenols extracted using 80% methanol and 80% ethanol from olive mill wastewater samples collected at collection pits (CP) and decanter (B1 and B3 on Figure 1).

The highest concentrations of total phenols were extracted from samples taken from a horizontal decanter (B1 in Figure 1), which is expected given that this wastewater is more concentrated and not diluted with technical water. However, significant TPC were also detected in samples from collection pits (symbol A, Figure 1), with concentrations reaching up to 3000 mg GAe L^−1^ of OMW. Methanol and ethyl acetate are among the most commonly used solvents for phenolic extraction due to their polarity and extraction efficiency [27,38,39]. Nonetheless, their toxicological effects are well documented, making ethanol a more environmentally friendly and safer alternative solvent. Despite methanol’s higher polarity, this study observed no statistically significant difference in phenolic compound extraction efficiency between 80% methanol and 80% ethanol (Wilcoxon signed rank test; W = 1449.0, *p* = 0.244) (Figure 2).

**Figure 3:**
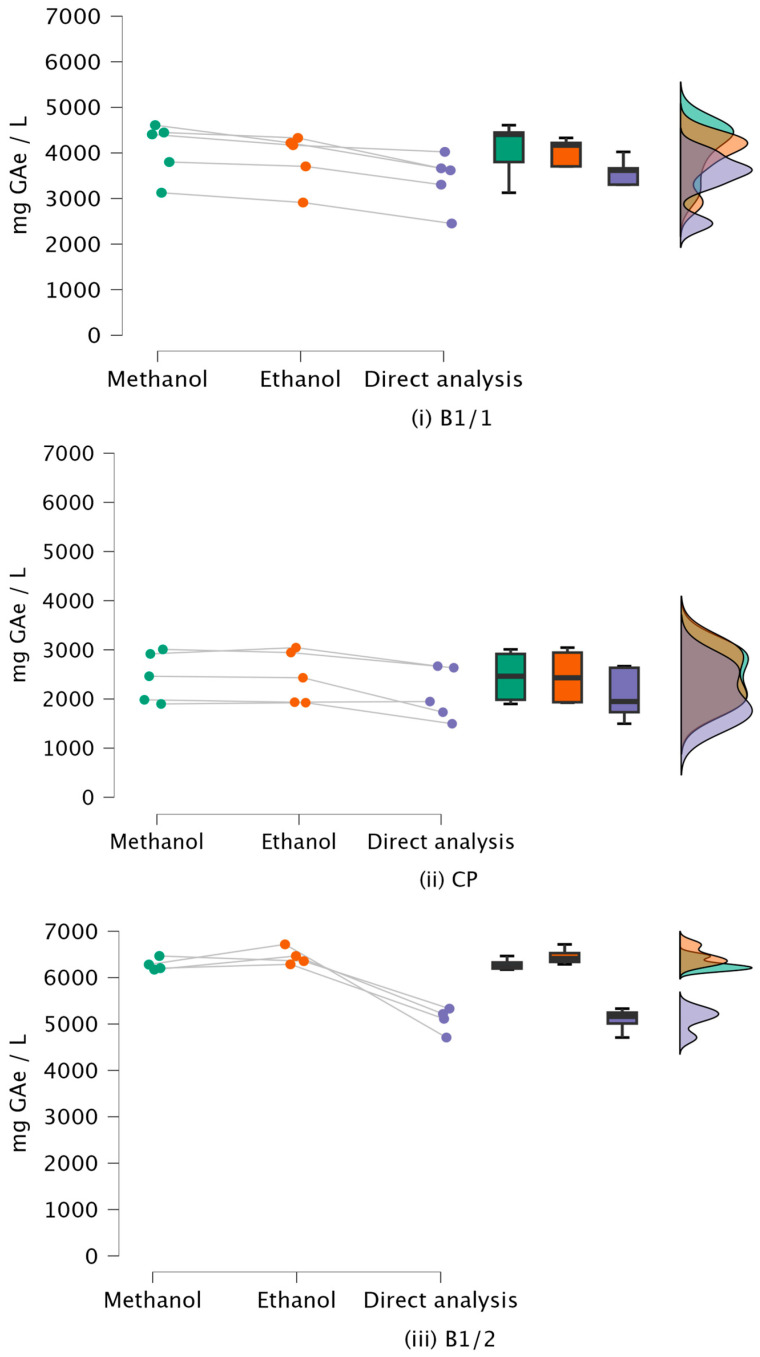
Total phenolic content (TPC, mg GAe L^−1^ OMW) in olive mill wastewater samples (*n* = 5 per group) obtained by extraction with 80% methanol and 80% ethanol by direct analysis after centrifugation and filtration for sampling locations B1/1 (**i**), CP (**ii**), and B1/2 (**iii**). Connected data points represent paired measurements of the same sample analyzed by the three methods, allowing visual comparison of within-sample differences across analytical approaches (color coded: methanol—green; ethanol—orange; direct analysis—blue).

Since polyphenols are water-soluble, the potential for direct determination of TPC after centrifugation alone was investigated. Fifteen OMW samples were selected to compare polyphenol quantification results obtained by solvent extraction (with 80% methanol and 80% ethanol) and by direct analysis following centrifugation and filtration (Figure 3). Ten samples were collected from two mills employing 3PD (B1 on Figure 1), and five samples originated from collection pits (CP) (location A on Figure 1).

A comparison of total phenolic content values obtained by solvent extraction and direct analysis across the different sampling locations is presented in Figure 3.

For the subset of samples collected at a specific point (*n* = 5), differences between the three analytical approaches (methanol extraction, ethanol extraction, direct analysis) were evaluated using a repeated-measures procedure on ranked data with Conover’s post hoc test and adjustment for multiple comparisons.

In the B1/1 samples (*n* = 5), there was a statistically significant difference in TPC between the three analytical approaches (Friedman test, χ^2^ = 12.00, df = 2, *p* = 0.002, Kendall’s W = 1.000). Post hoc Conover comparisons with Holm adjustment showed that methanol extraction yielded significantly higher TPC than ethanol extraction and direct analysis (all *p* < 0.001), while ethanol extraction also provided significantly higher TPC than direct determination (all *p* < 0.001).

In the CP samples (*n* = 5), there was a statistically significant difference in TPC between the three analytical approaches (Friedman test, χ^2^ = 7.00, df = 2, *p* = 0.030, Kendall’s W = 0.583). At the unadjusted level, Conover’s pairwise comparisons indicated significant differences between methanol extraction and direct determination (*p* = 0.004) and between ethanol extraction and direct determination (*p* = 0.034), whereas the difference between methanol and ethanol extraction was not significant (*p* = 0.249). After controlling for multiple testing using Holm adjustment, only the contrast between methanol extraction and direct determination remained statistically significant (p_Holm_ = 0.013).

In the B1/2 samples (*n* = 5), TPC differed significantly between the three analytical approaches (Friedman test, χ^2^ = 8.40, df = 2, *p* = 0.015, Kendall’s W = 0.840). Conover’s post hoc comparisons with Holm adjustment showed that both methanol and ethanol extraction yielded significantly higher TPC than direct determination (all p_Holm_ < 0.006), whereas the difference between methanol and ethanol extraction did not reach statistical significance.

Direct water analysis of OMW samples resulted in lower measured polyphenol levels compared to extractions using organic solvents, indicating reduced extraction efficiency when no solvents are applied. This effect was especially pronounced in samples from one decanter site (B1/2), which contained very high concentrations of TP. The findings suggest that organic solvents such as methanol and ethanol, due to their polarity, are more effective at extracting polyphenols from OMW. The extraction yield using methanol ranged from 11 to 19% higher, and ethanol ranged from 14 to 21% higher compared to direct water extraction alone. However, the results indicate that direct determination, given the simplicity and speed of the procedure, can serve as a screening method for obtaining information on the total phenol content.

### 3.3. HPLC Determination of Phenolic Content

HPLC analysis of phenolic composition was conducted on a selected subset of fifteen OMW samples to identify the phenolic compounds present and their relative proportions. Ten samples were collected from two different three-phase olive mills (symbol B1 in Figure 1), selected due to their high TPC, and five samples originated from collection pits (symbol A, Figure 1) (Table 2), where values represent the mean of duplicate analytical measurements for each sample.

The results demonstrate that the phenolic profile of OMW is strongly influenced by the technological stage of sampling. The highest TPC and hydroxytyrosol concentrations observed in the B1 samples correspond with the enzymatic activity expected during the decanter stage, where mechanical disruption and β-glucosidase activity facilitate the hydrolysis of oleuropein derivatives. Notably, although oleuropein is found in high concentrations in olive oil [40,41,42], it was completely absent in all OMW samples analyzed. Conversely, hydroxytyrosol, which is present in relatively low amounts in olive oil, is widely recognized as a major secondary metabolite derived from oleuropein transformation during ripening, storage, and especially olive processing [43].

Samples from collection pits (symbol A, Figure 1) exhibited the lowest phenolic content, likely due to their aqueous nature and dilution with technical water, which reduces enzymatic activity exposure. Thyrosol was consistently present across all sample groups with moderate variation, reflecting its chemical stability.

Comparing the results of this study with other research, a similar polyphenol profile with a high content of hydroxytyrosol and tyrosol is observed [27,44,45]. However, our results show higher concentrations of total polyphenols, as well as the aforementioned phenolic alcohols. A possible reason is that the OMW samples were collected in the early harvest phase when the polyphenol content is higher. Nevertheless, it should be noted that the comparative studies were performed using different olive cultivars and under distinct pedoclimatic conditions, which are known to significantly influence polyphenol accumulation. Therefore, the observed differences in total polyphenol concentrations cannot be attributed solely to processing technology but also reflect cultivar- and environment-dependent variability.

### 3.4. Antioxidant Activity

**Figure 4:**
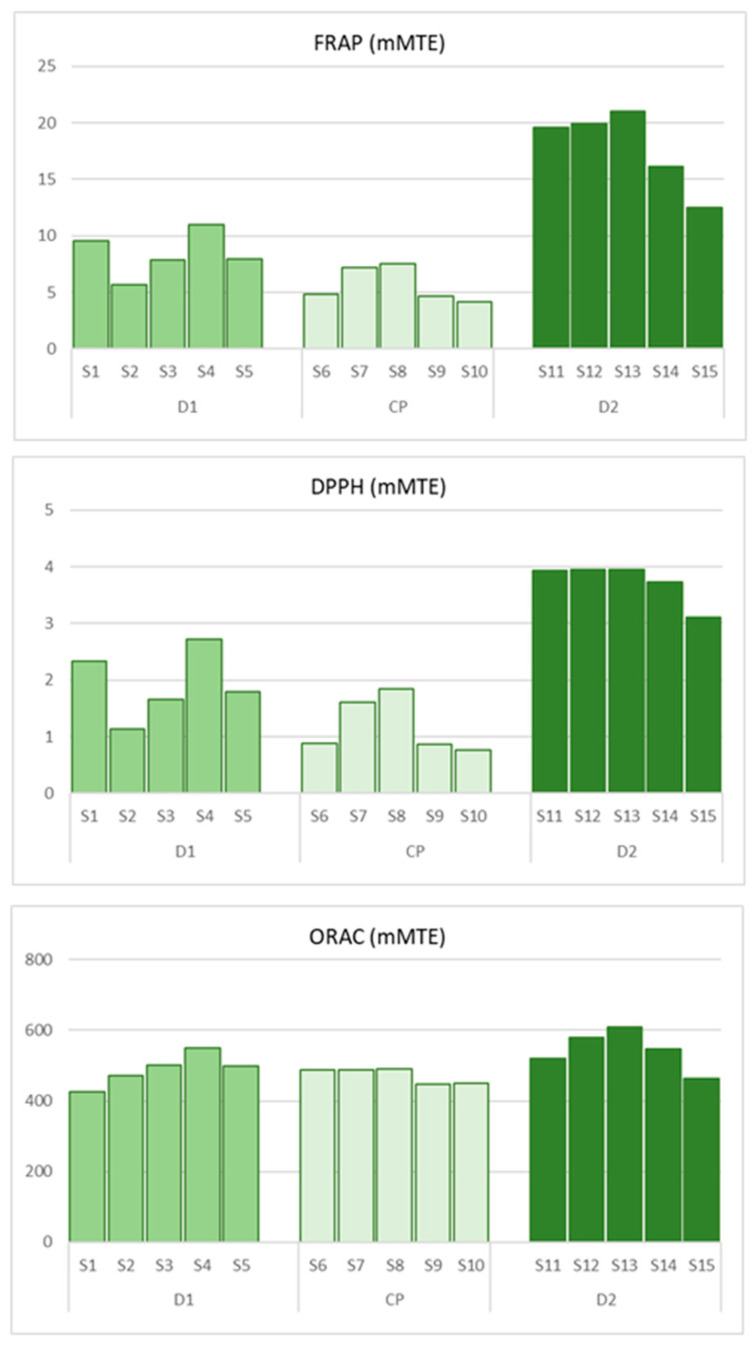
Antioxidant capacity (FRAP, DPPH, ORAC; mM TE) in fifteen individual OMW samples (S1–S15). Bars represent individual samples from decanter B1/1 (D1), collection pits (CP), and decanter B1/2 (D2), with values calculated as the mean of duplicate analytical measurements.

In the selected fifteen OWM samples, antioxidant capacity was assessed using three complementary assays (FRAP, DPPH, and ORAC), which revealed clearly significant differences between samples collected from collection pits and those from decanters (Table S1, Figure 4).

A Kruskal–Wallis test confirmed a statistically significant difference in FRAP values between locations (H = 11.58, df = 2, *p* = 0.003), and post hoc Dunn comparisons indicated that B1/2 had significantly higher FRAP values than CP (*p* < 0.001, p_Holm_ = 0.002), while other comparisons did not reach significance.

For DPPH, antioxidant capacity also significantly differed between sampling locations (H = 10.84, df = 2, *p* = 0.004). Dunn’s post hoc comparisons showed that samples from decanter B1/2 had significantly higher DPPH values than both B1/1 (*p* = 0.040, p_Holm_ = 0.080) and the collection pit A (p_Holm_ = 0.003), whereas the difference between B1/1 and A was not statistically significant (p_Holm_ = 0.229).

For ORAC values, the Kruskal–Wallis test did not reveal a statistically significant overall difference between locations (H = 4.56, df = 2, *p* = 0.102), indicating ORAC values were broadly similar across the three sampling points.

Wastewater from decanter B1/2 exhibited the highest antioxidant potential across all assays, with FRAP values ranging from 12.51 to 21.02 mM TE, DPPH inhibition between 3.10 and 3.95 mM TE, and ORAC values from 463.2 to 610.2 mM TE. This indicates a high content of phenolic compounds and a strong capacity to neutralize free radicals, including prolonged protection against peroxyl radicals. In contrast, samples from collection pits (symbol A, Figure 1) demonstrated significantly lower antioxidant capacities in the FRAP (4.17 to 7.55 mM TE) and DPPH (0.76 to 1.84 mM TE) assays compared to wastewater from decanter B1/2. However, ORAC results (446.7 to 490.3 mM TE) suggest the presence of stable hydrophilic phenolics in these samples (Figure 4), consistent with their ability to provide antioxidant protection, even though to a lesser extent relative to decanter samples.

Wastewater from decanter B1/1 exhibited moderate antioxidant capacity with FRAP values ranging from 5.69 to 10.95 mM TE, DPPH inhibition between 1.14 and 2.72 mM TE, and ORAC values from 426.0 to 551.4 mM TE. These results confirm that different processing stages significantly affect the transfer and concentration of phenolic compounds in wastewater. The combined use of the three assays provided a comprehensive evaluation of the antioxidant profile: FRAP and DPPH assays primarily reflect total phenolic content and reactivity toward free radicals, while ORAC assay additionally quantifies the kinetics and stability of the antioxidant effect.

These findings highlight the potential of olive processing wastewater as a valuable source of antioxidants and emphasize the need to optimize technological parameters during processing to maximize phenolic compound preservation and antioxidant potential.

The analysis of phenolic compounds (Table 2) and antioxidant activity (Table S1) in OMW demonstrates a distinct association between phenolic composition and functional antioxidant potential across processing stages. Wastewater samples from decanters B1/1 and particularly B1/2 exhibited elevated total phenolic content, largely dominated by hydroxytyrosol, while tyrosol, vanillic, caffeic, and cinamic acids contributed to both rapid radical scavenging activity, as measured by FRAP and DPPH assays, and prolonged antioxidant protection assessed by ORAC. In contrast, OMW collected from storage pits displayed lower TPC compared to decanter OMW, but maintained measurable antioxidant potential, likely due to the persistence of hydrophilic phenolic constituents. Overall, these findings indicate that both the concentration and compositional diversity of phenolic compounds critically influence the antioxidant potential of OMW, with hydroxytyrosol serving as the primary radical scavenger and secondary phenolics supporting sustained antioxidant effects.

### 3.5. Polyphenol Content Depending on the Type of Decanter

During the 2025 harvest season (October and November), a total of 104 OMW samples were collected from three types of oil mills at different discharge points. Sampling locations are marked in Figure 1, and they included 19 samples from B1 (horizontal decanter, 3-phase mill), 11 samples from B2 (vertical decanter, 2.5-phase mill), 62 samples from B3 (vertical decanter, 2-phase mill), and 12 samples from C (vertical decanter, 3-phase mill). The total phenols were determined after ethanol extraction according to a previously described protocol, since direct determination from water yields lower results, and methanol, although yielding slightly higher recovery, poses environmental and health concerns.

**Figure 5:**
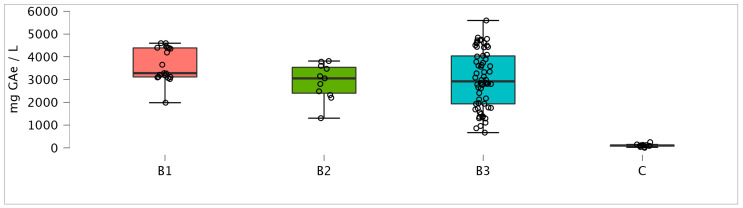
Total phenol content depending on sampling location; B1—horizontal decanter in 3-phase mill; B2—vertical decanter in 2.5-phase mill; B3—vertical decanter in 2-phase mill and C—vertical decanter in 3-phase mill.

The polyphenol content in OMW varies significantly depending on the type of oil mill extraction system and decanter used as shown in Figure 5. In a 3-phase oil mill, after horizontal centrifugation, two types of OMW are produced. The first type of wastewater (sample B1), collected immediately after horizontal centrifugation, is rich in suspended organic matter, giving it a denser texture and resulting in high PC, with median concentrations 3282.0 mg GAe L^−1^ OMW (IQR 3116.3–4390.2). Conversely, samples from the vertical decanter in 3PD have a very low polyphenol concentration (median 106,1 mg GAe L^−1^) due to the fact that most OMW in 3PD technology is separated after the horizontal decanter and a small amount flows to the vertical one, where it is further diluted with water.

In contrast, oil mills that operate in 2.5-phase or 2-phase systems (samples B2 and B3) are characterized by a smaller addition of water to the decanters compared to 3-phase which, considering the hydrophilic character of polyphenols, could indicate lower concentrations. However, the concentrations of polyphenols in these samples are not significantly lower compared to the samples from 3PD (sample B1) with TPC in samples from 2,5PD and 2PD mills (samples B2 and B3) with median values 3052.2 (IQR 2401.8–3537.6) and 2922.1 mg GAe L^−1^ (IQR 1933.5–4039.4).

Comparable investigations have been conducted in countries with well-developed olive-growing sectors, which similarly generate substantial quantities of potentially valuable by-products, particularly OMW. Several studies from Italy have reported lower OMW derived from three-phase decanters than those observed in the present study. Specifically, Cuffaro et al. reported TPC values ranging from 1529 to 457.8 mg GAe L^−1^ in OMW collected from a three-phase milling system, with higher concentrations measured at the beginning of the processing season compared with its end [46].

In the present study, OMW samples were collected at the onset of the harvest, when predominantly green or slightly black-streaked olives were processed, which likely contributed to the higher TPC values. In addition to the cultivar-dependent variability, TPC also depends on the ripeness of the fruit, with polyphenol content generally decreasing as the maturity index increases [47,48]. Similarly, Russo et al. and Girometti et al. reported TPC values of 1304 mg GAe L^−1^ and 501 mg GAe L^−1^, respectively [45,49]. In both studies, OMW samples were obtained from three-phase milling system; however, the precise sampling points within the processing line were not specified.

The findings of the present study highlight the importance of sampling location, revealing marked differences between horizontal and vertical decanters, as well as between two-phase and three-phase processing systems. In studies focusing on the recovery and potential exploitation of polyphenols from OMW, Kalogerakis et al. [50] and Athanasiadis [51] reported TPC values of approximately 3500 mg L^−1^, which are comparable to those observed in the present study.

Furthermore, studies conducted in Spain on abandoned evaporation ponds, where highly concentrated OMW (often referred to as alpechín) had accumulated over prolonged periods, reported exceptionally high polyphenol concentrations. Due to prolonged natural evaporation and the absence of dilution, TPC values in these systems reached up to 16 g GAe L^−1^, far exceeding those typically reported for fresh OMW streams [52].

Collectively, those findings provide compelling evidence that OMW represents a rich and consistent source of bioactive phenolic compounds across diverse production contexts. From fresh OMW streams in three-phase mills to highly concentrated alpechín in abandoned evaporation ponds, elevated levels of phenolic antioxidants have been consistently documented. This reinforces the perspective that OMW should be regarded not merely as an environmental burden but as a valuable resource with significant potential for sustainable exploitation in food, pharmaceutical, and cosmetic applications.

## 4. Conclusions

The aim of this work was to determine the polyphenol content in OMW generated by oil mills in the Dalmatian region, for which no published data were previously available.

Different extraction approaches were evaluated, including methanol and ethanol extraction as well as direct analysis of OMW. Considering both extraction efficiency and environmental sustainability, ethanol proved to be the most suitable solvent, yielding satisfactory results while offering a more environmentally friendly alternative.

OMW samples obtained from different types of oil mills were compared (three-phase, two-phase, and hybrid 2.5-phase). The highest TPC was observed in OMW collected from decanters in a 3-phase oil mill. Nevertheless, samples from the other two types of decanters also exhibited substantial polyphenol levels, confirming their significant bioactive potential.

The HPLC analysis revealed hydroxytyrosol and tyrosol as the predominant phenolic compounds in the OMW samples, which is consistent with the strong antioxidant activity observed in the corresponding extracts.

Overall, the findings of this study clearly demonstrate that OMW from mills in the Dalmatia region should be regarded not merely as an environmental burden, but as a valuable raw material and a promising source of natural antioxidants with potential applications in food, pharmaceutical, and cosmetic industries.

While the present study was intentionally designed to provide a regional overview and comparative evaluation of extraction approaches, several aspects merit further investigation to extend the applicability of these findings. In particular, future studies may benefit from incorporating well-defined cultivar-specific sampling and multi-seasonal datasets, as polyphenol composition in olive mill wastewater can be influenced by agronomic and pedoclimatic factors. Furthermore, although ethanol-based extraction proved effective and environmentally sustainable at the laboratory scale, subsequent research should focus on the optimization, validation, and techno-economic assessment of scalable extraction and purification processes. Additional investigations addressing the stability, safety, bioavailability, and performance of recovered polyphenols in real food, pharmaceutical, or cosmetic formulations would further support the industrial valorization of olive mill wastewater as a sustainable, high-value resource.

## Figures and Tables

**Table 1:** Total phenolic content (TPC) expressed as mg gallic acid equivalents per litre of olive mill wastewater (mg GAe L^−1^ OMW), obtained after extraction with 80% (*v*/*v*) methanol and 80% (*v*/*v*) ethanol from samples collected at collection pits (symbol A, Figure 1) and vertical decanter samples (symbol B1, Figure 1) during the 2023–2024 harvest season.

Harvest Year (Nb. of Samples)	Sampling Location (Figure 1)	Methanol		Ethanol		Methanol	Ethanol
		Median	IQR	Median	IQR	Concentration range *	
2023 (CP) (N = 24)	A	1147.7	745.6–1466.6	1261.8	779.5–1457.3	156.8–2647.8	205.8–2794.7
2024 (CP) (N = 37)	A	1042.4	705.7–1641.9	997.3	632.2–1665.8	417.8–3008.8	446.9–3045.2
2024 (D) (N = 21)	B1	4447.8	4346.3–6085.0	4398.8	4169.6–6193.4	3126.9–6466.9	2911.7–6717.2

* minimum–maximum observed values expressed as mg GAe L^−1^ OMW; CP-collection pit; D-decanter.

**Table 2:** Total phenolic content (TPC, mg GAe L^−1^ OMW) and concentrations of main polyphenolic compounds (mg L^−1^) in fifteen selected OMW samples. Values are presented as mean concentrations per sample, calculated from duplicate analytical measurements (technical replicates); no group means are reported. Sampling locations B1 and A correspond to those shown in Figure 1.

Polyphenolic Compound	Samples from Decanter B1/1				
	Sample 1	Sample 2	Sample 3	Sample 4	Sample 5
TPC	3704.5	2911.7	4219.6	4328.9	4169.57
Hydroxytyrosol	981.33	614.89	1163.97	1368.19	1366.47
Tyrosol	160.52	135.24	148.31	164.69	184.84
Oleoeuropein	n.d.	n.d.	n.d.	n.d.	n.d.
3,4-hydroxybenzoic acid	48.02	10.34	4.23	3.81	4.50
Vanillic acid	4.22	10.79	17.82	16.56	15.70
Caffeic acid	9.55	10.76	12.76	16.51	22.93
p-Coumarin acid	n.d.	n.d.	n.d.	n.d.	n.d.
t-Ferulic acid	6.65	10.90	6.53	14.82	15.91
o-Coumarin acid	6.27	3.21	n.d.	3.04	5.94
Cinamic acid	n.d.	3.50	n.d.	n.d.	n.d.
	**Samples from Collection pits A**				
	**Sample 6**	**Sample 7**	**Sample 8**	**Sample 9**	**Sample 10**
TPC	1934.0	3045.2	2944.3	1924.4	2431.6
Hydroxytyrosol	474.06	890.06	715.28	327.25	399.42
Tyrosol	128.27	240.98	198.11	86.08	70.07
Oleoeuropein	n.d.	n.d.	n.d.	n.d.	n.d.
3,4-hydroxybenzoic acid	56.45	n.d.	n.d.	n.d.	n.d.
Vanillic acid	3.17	9.48	6.95	n.d.	n.d.
Caffeic acid	n.d.	n.d.	n.d.	n.d.	8.34
p-Coumarin acid	14.09	21.03	30.05	16.26	3.56
t-Ferulic acid	1.71	n.d.	5.38	n.d.	2.48
o-Coumarin acid	2.00	3.18	8.91	2.38	1.17
Cinamic acid	2.82	2.66	3.26	2.88	3.06
	**Samples from Decanter B1/2**				
	**Sample 11**	**Sample 12**	**Sample 13**	**Sample 14**	**Sample 15**
TPC	6355.6	6287.2	6466.6	6717.2	6583.8
Hydroxytyrosol	2718.44	2610.28	2247.88	2174.48	1590.52
Tyrosol	201.48	262.42	186.70	358.16	227.42
Oleoeuropein	n.d.	n.d.	n.d.	n.d.	n.d.
3,4-hydroxybenzoic acid	n.d.	n.d.	n.d.	n.d.	n.d.
Vanillic acid	31.42	18.02	20.28	12.36	11.82
Caffeic acid	16.84	12.52	n.d.	8.06	6.18
p-Coumarin acid	n.d.	n.d.	n.d.	n.d.	n.d.
t-Ferulic acid	n.d.	n.d.	n.d.	n.d.	n.d.
o-Coumarin acid	n.d.	n.d.	n.d.	n.d.	n.d.
Cinamic acid	10.50	19.82	20.66	16.52	21.22

n.d. not detected.

## Data Availability

The data presented in this study are available upon request from the corresponding author.

## Supplementary Materials

The following supporting information can be downloaded at: https://www.mdpi.com/article/10.3390/antiox15010012/s1, Table S1. Antioxidant capacity of selected olive mill wastewater samples measured by FRAP, DPPH and ORAC assays. Results are expressed as mean ± standard deviation of three replicate measurements in millimole Trolox equivalents (mM TE).
