# Supplementary material for: From Waste to Resource: Extraction and Characterization of Polyphenols from Dalmatian Olive Mill Wastewater
Source: Antioxidants (Basel). 2025 Dec 21;15(1):12. doi: 10.3390/antiox15010012 (PMC12838249; doi:10.3390/antiox15010012)
Supplement: Supplementary file 1 [file antioxidants-15-00012-s001.zip › antioxidants-4046559-supplementary.pdf]

Table S1. Antioxidant capacity of selected olive mill wastewater samples measured by FRAP, DPPH and ORAC assays. Results are expressed as mean ± standard deviation of three replicate measurements in millimole Trolox equivalents (mM TE).

	**Samples from B1/1**				
	Sample 1	Sample 2	Sample 3	Sample 4	Sample 5
FRAP (mM TE)	9.56 ± 0.36	5.69 ± 0.28	7.90 ± 0.04	10.95 ± 0.12	7.96 ± 0.08
DPPH (mM TE)	2.33 ± 0.03	1.14 ± 0.08	1.66 ± 0.04	2.72 ± 0.05	1.79 ± 0.06
ORAC (mM TE)	426.0 ± 20.78	472.2 ± 60.72	500.3 ± 44.94	551.4 ± 34.77	498.8 ± 23.47
	**Samples from A**				
	Sample 6	Sample 7	Sample 8	Sample 9	Sample 10
FRAP (mM TE)	4.81 ± 0.13	7.17 ± 0.25	7.55 ± 0.19	4.63 ± 4.63	4.17 ± 0.04
DPPH (mM TE)	0.89 ± 0.10	1.60 ± 0.01	1.84 ± 0.04	0.87 ± 0.01	0.76 ± 0.07
ORAC (mM TE)	486.9 ± 17.84	488.5 ± 12.78	490.3 ± 33.58	446.7 ± 22.00	451.3 ± 12.97
	**Samples from B1/2**				
	Sample 11	Sample 12	Sample 13	Sample 14	Sample 15
FRAP (mM TE)	19.60 ± 0.38	19.88 ± 0.63	21.02 ± 7.61	16.15 ± 0.73	12.51 ± 0.29
DPPH (mM TE)	3.93 ± 0.01	3.95 ± 0.01	3.95 ± 0.01	3.73 ± 0.04	3.10 ± 0.05
ORAC (mM TE)	520.1 ± 28.85	578.9 ± 30.21	610.2 ± 33.26	548.5 ± 14.50	463.2 ± 13.66
